# Burnout syndrome among health care workers during the COVID-19 pandemic. A cross sectional study in Monastir, Tunisia

**DOI:** 10.1371/journal.pone.0282318

**Published:** 2023-03-23

**Authors:** Imen Zemni, Wafa Dhouib, Sihem Sakly, Cyrine Bennasrallah, Amel Gara, Meriem Kacem, Manel Ben Fredj, Hela Abroug, Aicha Elbaroudi, Ines Bouanene, Asma Sriha Belguith

**Affiliations:** 1 Department of Epidemiology and Preventive Medicine, Fattouma Bourguiba University Hospital, University of Monastir, Monastir, Tunisia; 2 Department of Epidemiology, Faculty of Medicine of Monastir, University of Monastir, Monastir, Tunisia; 3 Technology and Medical Imaging Research Laboratory—LTIM—LR12ES06, University of Monastir, Monastir, Tunisia; 4 Emergency Department, Ksar Hellal Hospital, Monastir, Tunisia; 5 Department of Occupational Health, Ksar Hellal Hospital, Monastir, Tunisia; National Cancer Institute of Brazil (INCA/MS), BRAZIL

## Abstract

**Background:**

Burnout syndrome may affect the safety of frontline healthcare care workers (HCW) and patients. We aimed to measure the prevalence of burnout among HCW in care facilities in Tunisia during the Covid-19 pandemic and to identify its associated factors.

**Methods:**

We conducted a cross-sectional study among HCW practicing during the covid-19 pandemic in health care facilities in the governorate of Monastir. Data collection was carried out using an anonymous self-administered questionnaire composed by three sections: epidemiological and clinical characteristics, professional conditions and the Maslach Burn out Inventory (MBI-HSS).

**Results:**

This study included 371 HCW. The prevalence of burnout was 77.9% (CI 95%: 73.6% - 82.1%). The severe level was found in 71 participants (19.1%), the moderate level in 115 (31%) and the low level in 103 (27.8%). The distribution of the levels of the burnout dimensions among the participants was as follows: high emotional exhaustion (EE) (57.4%), high depersonalization (DP) (39.4%) and low personal accomplishment (22.6%). The main determinants of burnout among healthcare professionals during COVID 19 pandemic were: working more than 6 hours per day (OR = 1.19; CI95% [1.06; 1.34]), physician function (OR = 1.17; CI 95% [1.05; 1.31]), feeling a negative impact of work on family life (OR = 1.40; 95% CI [1.13; 1.73]), and high personal estimation of COVID 19 exposure (OR = 1.15; CI95% [1.02; 1.29]).

**Conclusion:**

During the COVID19 pandemic, the prevalence of burnout among health professionals was high. It was related to hard implication in COVID 19 management. Interventions like adjusting working hours, reducing workload, and providing psychological support should be taken.

## Background

The COVID-19 pandemic resulted in higher mortality than previous viral pandemics of the past four decades [1]. Health professionals were on the front line to manage this pandemic in both screening and managing positive patients regardless of their severity. Since psychological suffering may be associated with the uncertainty of a safe workplace, the World Health Organization (WHO), has emphasized, during the COVID-19 pandemic, the importance of improving the mental health and psychological well-being of health-care workers (HCW) [2]. Burn-out is a syndrome conceptualized as resulting from chronic workplace stress and defined as emotional exhaustion, depersonalization, and low personal achievement [3]. The prevalence and factors associated with burnout, differed according to the socioeconomic level of the country and the culture of HCW [4]. Overcrowding in hospitals and intensive care units (ICUs) and shortages of basic equipment and consumables have led to an increased burden of COVID-19 on health systems and a greater physical and psychological impact on health care personnel, particularly in LMICs where the prevalence of burnout ranges from 2.5% to 87.9% [5, 6]. In order to manage this syndrome, regardless of the pandemic, studies have shown a range of factors associated with burnout have been reported, including demographics, heavy workload, second job, inadequate exposure to resources, inadequate personal protective equipment(PPE), low level of support, job insecurity, specific job tasks, inadequate breaks or vacation time, and years of service [6, 7]. In these countries, the few studies that were conducted during the COVID-19 pandemic found that increased exposure to COVID-19 patients, working in the frontlines and PPE shortages were both positively associated with burnout [8, 9]. Tunisia, one of the developing countries, has been largely affected by the COVID-19 pandemic. As of January 11, 2022, the total number of COVID-19 cases has reached 756,155, with a case fatality rate of 3.4%. The inpatient rate in public and private hospitals was 10.3%, and about a quarter of patients were in ICU [10]. As a result, Tunisia has undergone an overload and saturation [11] of the health system without sufficient financial resources to protect the health care personnel.

Although a few Tunisian studies have shown that some caregivers may suffer from psychological impact such depression, anxiety, and insomnia [12–14], no study has yet looked at burnout among health workers during this pandemic.

We aimed to measure the prevalence of burnout among HCW in care facilities in the Monastir region during the Covid-19 pandemic and to identify factors associated with this burnout.

## Methods

### Study design

This is a cross-sectional study conducted among health care professionals at health facilities in the Monastir region during the Covid-19 pandemic between November 2020 and October 2021.

### Study setting

Monastir Governorate is one of the twenty-four governorates of Tunisia. It is situated in the east of Tunisia. It is one of the four cities (Tunis, Sousse, Monastir and Sfax) with a hospitalo-university vocation in the country. The total number of health professionals in Monastir is 914 physicians and 2,247 paramedical personnel. Health facilities are divided into three levels: primary, secondary, and tertiary. According to the latest update of the health card of 2019, the main state tertiary-level structure in Monastir is "Fattouma Bourguiba University Hospital ". Five other small private clinics are also tertiary. Besides, the governorate has two secondary-level structures: the Regional Hospital of Moknine and the Regional Hospital of Ksar Hellal. The other structures are primary and consist of nine constituency hospitals and 98 basic health centers.

### Study population

All health professionals practicing in tertiary, secondary, and primary health care facilities in the Monastir region were included in the study. Medical professions included medical doctors (Physicians, dentists, and medical residents). Paramedical professions included nurses, anesthesia technicians, medical biology technicians, physiotherapists, dieticians, paramedics. . .

The required sample size was calculated based on a prevalence rate of burnout syndrome of 84% [15] using the following formula: *n* ₌ (*z*_α/2_)^2^ $\frac{p(1-p)}{i^{2}}$ with *[z*_*α/2*_ *₌ 1*,*96*, *i₌ 0*.*05*, *p ₌ estimated prevalence]*. The minimum number of subjects required was then 207 health professionals.

### Data collection and variable definitions

Data collection was conducted using an anonymous self-administered questionnaire. The survey questionnaire covered demographic characteristics (Age, gender, marital status, having children, chronic condition and history of Covid-19 infection), work conditions (Institution, professional category, work experience, work schedule, number of shifts per week, estimated exposure to Covid-19 during regular clinical activity, self-assessment of the effectiveness of personal protective equipment in the institution, department, working in a department that hospitalizes Covid-19 patients, close contact with Covid-19 patient…) and the French version of Maslach Burn out Inventory (MBI-HSS) [16] to assess the level of burnout. This psychometric instrument consists of 22 items evaluating three dimensions of burnout: Occupational exhaustion (EE) (nine items): 1, 2, 3, 6, 8, 13, 14, 16, 20; Depersonalization (DP) (five items) 5, 10, 11, 15, 22 and Personal accomplishment assessment (PA) (eight items): 4, 7, 9, 12, 17, 18, 19, 21. The total score of each dimension indicates a high, moderate or low level as mentioned in Table 1 [17]. Burnout occurs if EE high, or DP high or PA low. The degree of burn out is specified as follows [16, 17]: **1. Low burnout** if only one dimension is affected: high EE or high DP or low PA. **2. Moderate burnout** if two dimensions are affected (High EE + High DP), or (High EE + Low PA) or (High DP + Low PA). **3.• Severe burnout** if all three dimensions are affected: (High EE + High DP + Low PA).

**Table 1:** Maslach burnout inventory interpretation.

	High	Moderate	Low
**Occupational exhaustion (EE)**	> 29	18–29	<18
**Depersonalization (DP)**	>11	6–11	< 6
**Personal accomplishment assessment (PA)**	<34	34–39	> 39

The reliability of the Maslach Burn out Inventory was assessed using Cronbach’s Alpha Coefficient, which was 0.75 indicating the sufficient level of reliability. Cronbach’s Alpha Coefficient was 0.87, 0.69, and 0.76, respectively, for Occupational exhaustion (EE), Depersonalization (DP) and Personal accomplishment (PA).

### Data analysis

Data were verified and analyzed using IBM SPSS Statistics version 22.0 software. Qualitative variables were represented by numbers and percentages, quantitative variables by their means and standard deviations. To identify associated factors with burnout, univariate analysis was established using the chi-square test. The determinants of burn out were identified through multivariate analysis using Poisson regression with robust variance. The threshold for statistical significance was set at 5%.

### Ethical considerations

The study was conducted under Good Clinical Practice conditions and according to ethical standards collections. Written informed consent was obtained from all participants. Protection of the privacy of research subjects as well as confidentiality of their personal information was ensured.

## Results

### General and occupational characteristics

This study included 371 health professionals. The average age of the study population was 36.65 years (SD:8.95), with extremes of 21 and 62 years. Female sex was predominant (79.5%). More than two-thirds of this population felt that their function as a health professional had a negative impact on their family life. Prevalence of Covid-9 infection was 35.8% (CI95%: 30.9% - 40.6%). Paramedical staff and medical residents represented the two main professional categories with respective proportions of 48% and 20.2%. Among participants, 64.4% cared for COVID-19 patients as part of their clinical activities and 47.5% considered their exposure to COVID-19 to be high, but only 42.3% felt adequately protected by personal protective equipment (PPE) in their institutions. The general and occupational characteristics of the study population are described in Table 2.

**Table 2:** General and occupational characteristics of the study population.

		N = 371	
***Gender, n (%)***	Male	76	(20.5)
	Female	295	(79.5)
***Age (years), mean (SD)***		36.67	*(8*.*95)*
***Age classes(years), n (%)***	< 30	90	(24.3)
	[30–40]	165	(44.5)
	[40–50]	71	(19.1)
	≥ 50	45	(12.1)
***Marital status, n (%)***	Single	104	(28.0)
	Married	256	(69.0)
	Divorced	9	(2.4)
	Widower	2	(0.5)
***Have children***	No	128	(34.5)
	Yes	243	(65.5)
***Chronic conditions, n (%)***	No	168	(45.3)
	Yes	56	(15.1)
	Missing	147	(39.6)
***Previous infection with Covid19, n (%)***	No	238	(64.2)
	Yes	133	(35.8)
***Feeling a negative impact of work on family life, n (%)***	No	73	(19.7)
	Yes	298	(80.3)
***Work experience (years), mean (SD)***		9.55	8.69
***Work experience classes(years), n (%)***	≤ 5	168	(45.3)
	[5–10]	69	(18.6)
	[10–20]	82	(22.1)
	>20	45	(12.1)
	Missing	7	(1.9)
***Health facility, n (%)***	University hospital	107	(29.1)
	Regional hospital	134	(35.3)
	Constituency hospital / BHC	94	(25.3)
	Private	37	(10.0)
	Missing	1	(0.3)
***Professional category, n (%)***	Nurses	128	(34.5)
	Other paramedical professions	60	(16.2)
	General practitioner	53	(14.6)
	Medical resident	76	(20.2)
	Academic physician	21	(5.4)
	Dentist	34	(9.2)
***Department, n (%)***	Intensive care units/Emergency	119	(32.1)
	Covid19 units	22	(5.9)
	Medical department	85	(22.9)
	Surgical department	36	(9.7)
	Stomatology	35	(9.4)
	Primary health level	34	(9.1)
	Others	40	(17.8)
***Working in a department hospitalizing Covid19 patients, n (%)***	No	154	(41.5)
	On shift	82	(22.1)
	Every day	101	(27.2)
	Missing	34	(9.2)
***Number of hours at work/day, n (%)***	6 H	195	(52.6)
	12 H	108	(29.1)
	18 to 24 H	68	(18.3)
***Number of shifts per week, n (%)***	No shifts	178	(48.0)
	1–2	132	(35.6)
	≥ 3	61	(16.4)
***Caring for Covid19 Patients, n (%)***	No	132	(35.6)
	Yes	239	(64.4)
***Personal estimation of Covid-19 exposure, n (%)***	Low	28	(7.5)
	Moderate	167	(45.0)
	High	176	(47.5)
***Have had close contact with a Covid-19 patient, n (%)***	No	71	(19.1)
	Yes	300	(80.9)
***Feel protected by PPE, n (%)***	No	213	(57.6)
	Yes	158	(42.6)

BHC: Basic Health Center PPE: Personal Protective Equipment

### Prevalence of burnout

**Fig 1:**
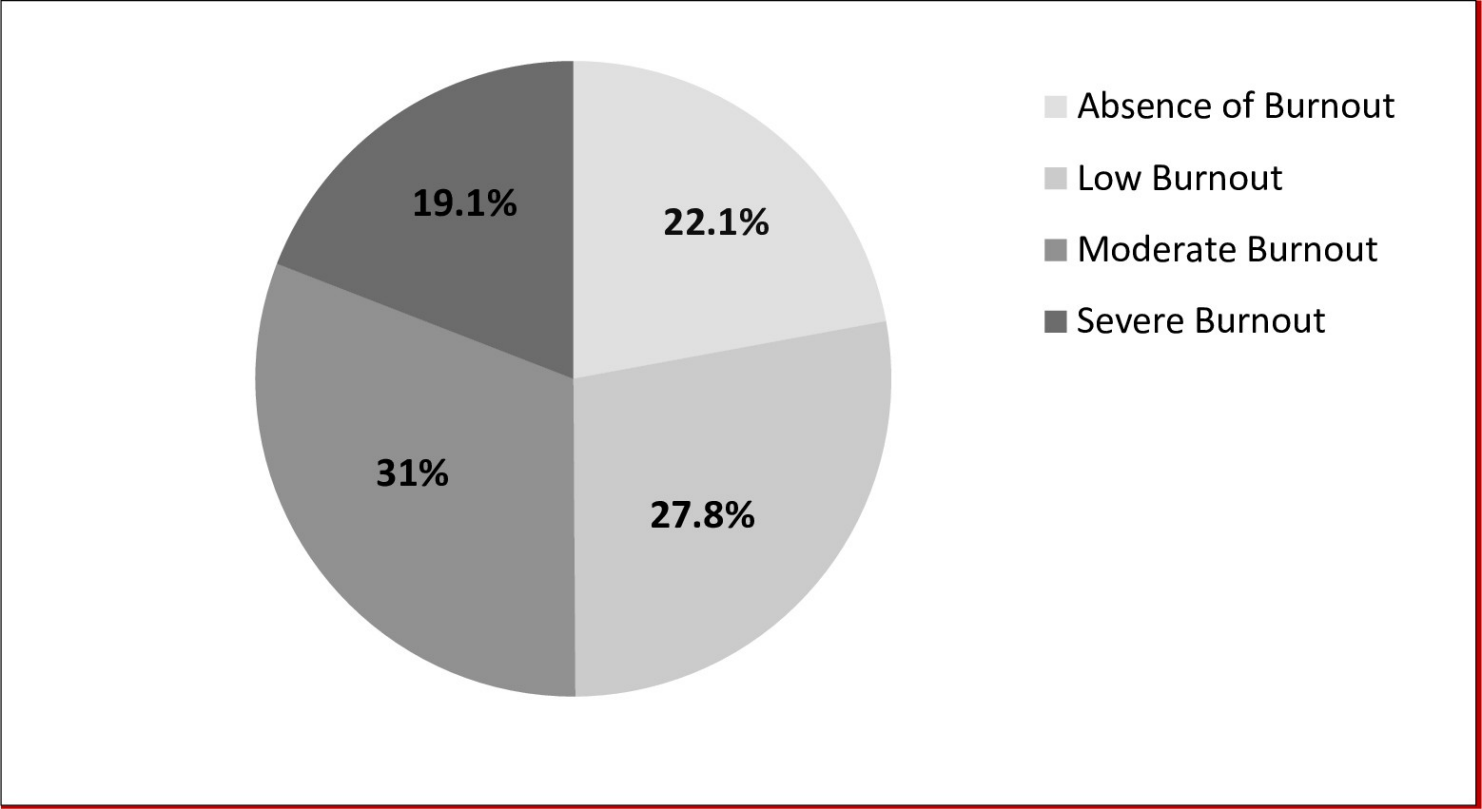
Distribution of the study population according to the level of burnout.

**Fig 2:**
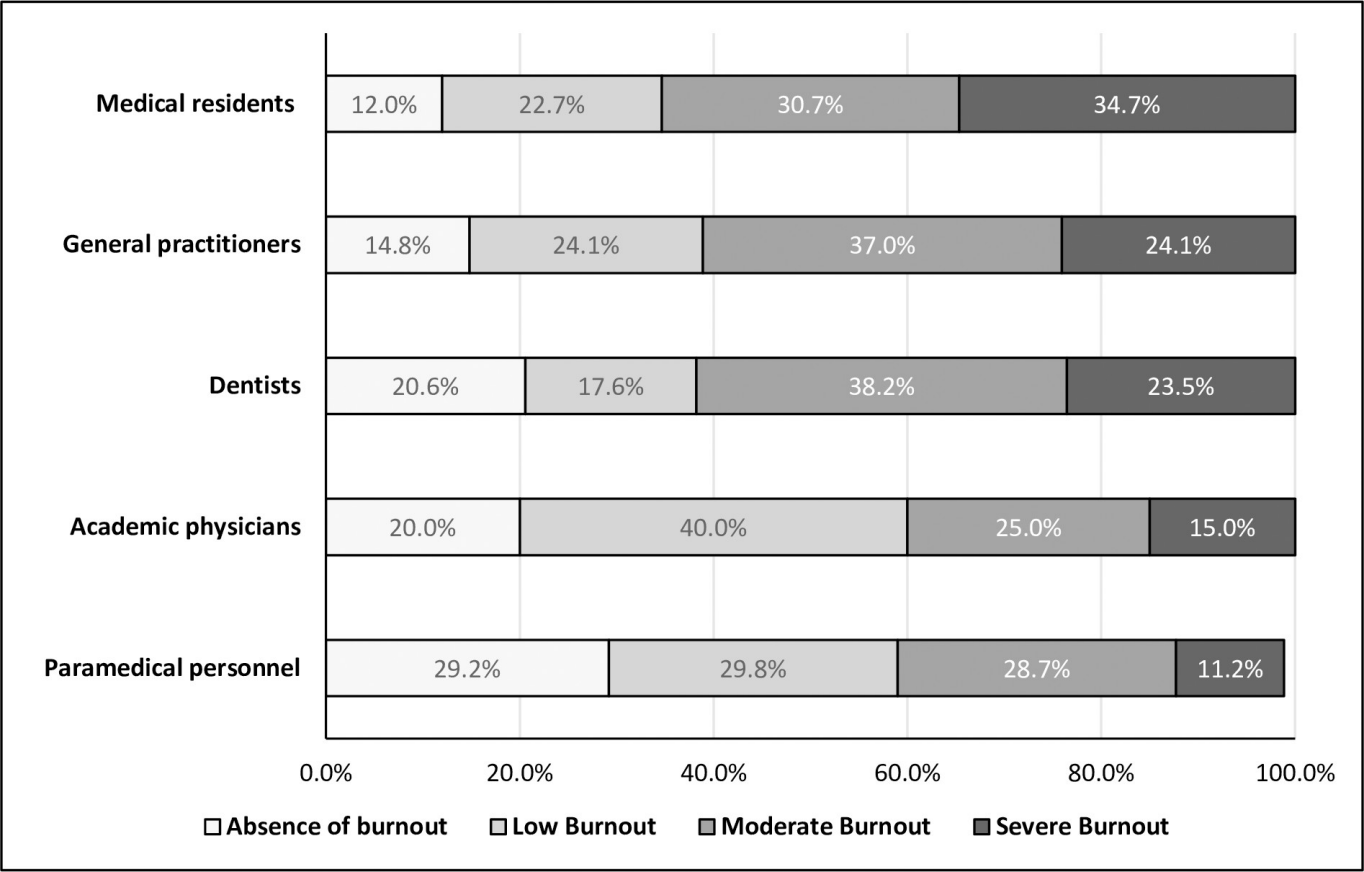
Levels of burnout according to the occupational status.

**Fig 3:**
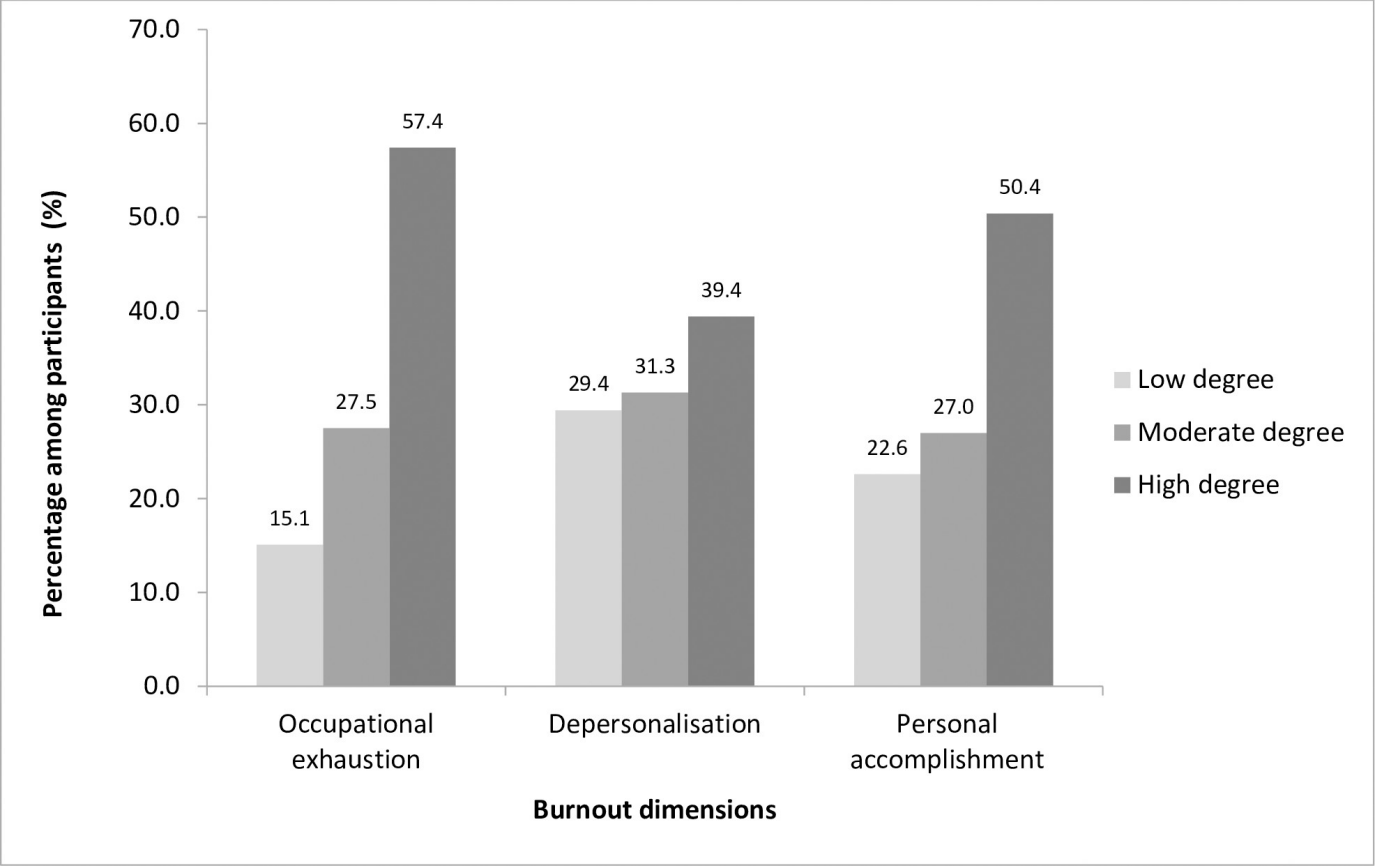
Distribution of burnout dimensions levels among participants.

Analysis of the Maslach Burn out Inventory results revealed that the majority of participants 289 (77.9%) (CI 95%: 73.6% - 82.1%) were in a burnout situation. The severe level, moderate level and low level were respectively recorded among 19.1% (CI95%: 15% -23.1%); 31% (CI95%: 26.2% -35.7%) and 27.8% (CI95%: 23.2% -32.3%) of participants (Fig 1). Severe burnout levels were mainly observed among medical residents (Fig 2). High levels of occupational exhaustion (EE), high levels of depersonalization (DP) and low personal accomplishment levels (PA) were recorded respectively among 57.4%, 39.4% and 22.6% of respondents (Fig 3).

### Associated factors with burnout

Factors associated with burnout in univariate analysis were: Working in Covid19 units and intensive care units (p = 0.045), daily working in a department that hospitalized positive Covid-19 patients (p = 0.007), medical professional categories (p = 0.001), the usual number of hours of work per day (p = 0.007), the number of shifts per week (p = 0.045), high personal estimate of exposure to covid-19 (p<10^-^^3^) and feeling a negative impact of work during the pandemic on family life (p<10^-^^3^) (Table 3).

**Table 3:** Associated factors with burnout among health professionals during the Covid19 pandemic in Monastir (Univariate analysis).

		N	Burnout		p
			n	%	
***Gender***	Male	76	59	77.6	0.95
	Female	295	230	78.0	
***Age Class (years)***	< 30	90	71	78.9	0.49
	[30–40]	165	133	80.6	
	[40–50]	71	151	71.8	
	≥ 50	45	34	75.6	
***Marital status***	Single	104	87	83.7	0.095
	Other	267	202	75.7	
***Have children***	No	128	106	82.8	0.098
	Yes	243	183	75.3	
***Chronic conditions***	No	168	120	71.4	0.79
	Yes	56	41	73.2	
***Previous infection with Covid19***	No	238	184	77.3	0.71
	Yes	133	105	78.9	
***Feeling a negative impact of work on family life***	No	73	41	56.2	**<10** ^**−3**^
	Yes	298	248	83.2	
***Work experience (years)***	≤ 5	168	138	82.1	0.08
	[5–10]	69	53	76.8	
	[10–20]	82	64	78.0	
	>20	45	29	64.4	
***Health facility***	University hospital	108	86	79.6	0.69
	Regional hospital	131	99	75.6	
	Constituency hospital / BHC	94	72	76.6	
	Private	37	31	83.8	
***Professional category***	Paramedical	183	134	71.3	**0.002**
	Medical	188	155	84.7	
***Department***	Covid19 units	22	20	90.9	**0.045**
	Intensive care units/Emergency	119	99	83.2	
	Others	230	170	73.9	
***Working in a department hospitalizing Covid19 patients***	No	154	108	70.1	**0.007**
	On shift	82	64	78.0	
	Every day	101	88	87.1	
	Oui pendant				
***Number of hours at work/day***	6 H	195	141	71.8	**0.007**
	12 H	108	94	87.0	
	18 to 24 H	68	55	80.9	
***Number of shifts per week***	No shifts	178	129	72.5	**0.045**
	1–2	132	111	84.1	
	≥ 3	61	49	80.3	
***Caring for Covid19 Patients***	No	132	97	73.5	0.12
	Yes	239	192	80.3	
***Personal estimation of Covid-19 exposure***	Low or moderate	195	138	70.8	**<10** ^**−3**^
	High	176	151	85.8	
***Have had close contact with a Covid-19 patient***	No	71	52	73.2	0.29
	Yes	300	237	79.0	
***Feel protected by PPE***	No	213	170	79.8	0.30
	Yes	158	119	75.3	

BHC: Basic Health Center PPE: Personal Protective Equipment

The multivariate analysis identified four determinants of burnout among health professionals during the covid19 pandemic (Table 4). These factors are working more than 6 hours per day, medical professional categories, feeling of a negative impact of work on family life, and high personal estimate of Covid-19 exposure with respective adjusted Odds Ratios (a OR) of 1.19; (CI95% = [1.06; 1.34])), 1.17 (CI95% = [1.05–1.31]), 1.40 (CI95% = [1.13–1.73]) and 1.15 (CI95% = [1.02–1.29]).

**Table 4:** Associated factors with burnout among health professionals during the Covid19 pandemic in Monastir (Multivariate analysis).

	Associated factor	Reference	Multivariate Analysis		
			p	OR	CI 95%
Professional category	Medical	Paramedical	0.004	1.17	[1.05–1.31]
Feeling a negative impact of work on family life	Yes	No	0.002.	1.40	[1.13–1.73]
Personal estimation of Covid-19 exposure	High	Low or Moderate	0.01	1.15	[1.02 – 1.29]
Number of hours at work/day	> 6H	≤ 6H	0.003	1.19	[1.06 – 1.34]

OR: Odds Ratio; CI: Confidence Interval

## Discussion

Our study clearly met its objectives by reporting a high prevalence of burn out during the COVID-19 pandemic with a severe level among medical residents. Predictors of burnout were medical occupational categories, a sense of negative impact of work on family life, a high personal estimate of Covid-19 exposure and excessive work hours.

This study highlights the significant impact of the Covid-19 pandemic on burn out of health care professionals, as it was higher (77.9%) than that found in previous studies in Tunisia or in other LMICs [18–20]. Currently, during this pandemic, this prevalence is within the range assumed in studies of burnout among primary care professionals in LMICs (2.5% to 87.9%) [6].

Indeed, the COVID-19 pandemic has increased workload, guilt, stigma, lack of PPE, and has become a new health care-associated disease that may exacerbate risk factors and promote mental ill health and thus burnout among HCW [21]. In the literature, there is a difference in the prevalence of burnout between countries depending on their economic level.It was higher than those conducted in France (55%) [22] and Iran (53%) [23] and lower than that conducted in Morocco (84.44%) [15]. A global study of 60 countries reported a 51% prevalence of burnout during the COVID-19 pandemic with the highest prevalence in the USA (62%) [8, 24]. This difference in prevalence between developed and developing countries could be explained by the pre-existing vulnerability of the health care system, which has been challenged by this pandemic, resulting in overwork and lack of protection for caregivers.

When considering the three dimensions of the MBI instrument, our results show high levels of of EE of 57.4%, high levels of DP of 39.4%, and low PA levels of 22.6%. These results were superior to those obtained in LMIC where the pooled prevalence of burnout among primary care professionals revealed a high level of EE of 28.1% (95% CI: 21.5–33.5), a high level of DP of 16.4% (95% CI: 10.1–22.9) and PA of 31.9% (95% CI: 21.7–39.1) [6].

According to the literature, these dimensions can account for the process of installation of burn out and therefore, the first phase of EE is already started in about half of the study population. EE reflects the emotional dimension of burnout and can be both physical and psychological, which could be explained by excessive workload and lead to reduced personal accomplishment [25].

According to our study, medical health personnel was 1.3 times more likely to develop burn out. As other Tunisian studies [13, 19, 20], it was clear that physicians, and in particular medical residents, were the most at risk of developing burnout syndrome. Our results were consistent with those obtained in Morocco, Iran, and the United States [16–18]. Several studies have highlighted the importance of this problem among physicians and explained that repeated exposure to a plethora of emotions, including the need to save the patient, feelings of failure and frustration as the patient’s illness progresses, feelings of helplessness in the face of illness and associated losses, bereavement, fear of becoming ill oneself or dying, uncertainty in clinical practice, and the experience of distress contribute to the high levels of stress that physicians experience in their profession [4]. Fekih et al explained that medical residents are functioning simultaneously as both learners and caregivers and experiencing considerable challenges during the pandemic [13].

Similar to our results, multiple studies have shown that higher personal estimates of COVID-19 exposure and increased contact time with COVID-19 patients were associated with higher scores on depression and burnout scales, with a pooled RR of 1.18, 95% CI = 1.05–1.32, p = .005) [24]. In a nationwide cross-sectional survey in Turkey, it was observed that the duration of contact with COVID-19 positive patients affected residents’ depression and burnout scale scores [8]. Likewise, it was observed that American medical trainees who were exposed to COVID-19 patients had higher prevalence of stress (29.4%), and burnout (46.3%) [26].

It has been reported in the literature that HCW working in front line and come into direct contact with suspected or confirmed cases of COVID-19 in hospitals are the most susceptible to this disease, and this situation has multiple negative effects on mental health [6, 9].

In two studies conducted in Turkey and Saudi Arabia, more than half of the physicians reported that their greatest concern about the pandemic was the fear of transmitting the virus to their families [8, 27].

Other factors of burnout, protective or not, have been found in the literature and have not been clearly identified in our study such as demographic factors (age, sex, marital and parental status.) [6]. As various studies [18, 28], we found no difference in the predictors of burnout according to age and sex. Others found a higher prevalence of burnout in women and suggest that this is due to their physical and psychological vulnerability, then to a greater emotionality towards the sick and finally, the difficulty of reconciling work and family life [15, 24]. Adequate PPE has been widely shown to protect against burnout (RR = 0.88, 95% CI = 0.79–0.97, p = 0.01) [24]; however, our study did not show an association. This could be explained by the fact that infection control with limited resources is a chronic problem that has been widely discussed in developing countries [29]. Factors associated with burnout in low- and lower-middle-income countries were the distance to work and having to perform tasks beyond the individual’s skills. Protective factors identified were exercise, breaks, and vacation time [6].

The study had some imitations. First, it is limited to HCW in Monastir governorate and results may not be generalised to all HCW in Tunisia. However, since Monastir is one of the main four cities with a hospitalo-university vocation in the country, the three levels of care (primary, secondary, and tertiary) as well as all categories of HCW are well represented. Second, this cross-sectional study evaluated burnout at a specific time, whereas prospective cohort studies could better evaluate the impact of this pandemic on health professionals.

In the light of our results, a set of preventive measures should be recommended to mitigate burn out during pandemics especially among categories with high risk of burn out (medical staff especially medical residents). Thus, interventions should include promoting a healthy work environment; limiting the maximum number of hours and days worked consecutively and ensuring work-life balance measures (hobbies, family, and social activities). Some practitioner-focused interventions may also be recommended such as stress reduction training and relaxation techniques. On the other hand, screening for burnout syndrome appears to be periodically necessary among HCW and especially those who have high exposure to COVID-19 patients.

## Conclusion

Our work has shed light on the prevalence and associated factors of burnout among health professionals in Tunisia during the covid-19 pandemic. This could help us to strengthen primary prevention strategies. These should be based on strengthening psychological training, adjusting working hours and reducing workload. Further investigations are needed to explore how HCWs´ burnout may affect the quality of care during a pandemic.

## Supporting information

S1 File Burnout syndrome among health care workers during the COVID-19 pandemic. A cross sectional study in Monastir, Tunisia.
(XLSX)

## Abbreviation

CI: Confidence Interval
DP: Depersonalization
EE: Emotional Exhaustion
HCW: Healthcare Care Workers
ICU: : Intensive Care Unit
LMICs: Low- and middle-income countries
MBI-HSS: Maslach Burn out Inventory Human services survey
OR: Odds Ratio
